# Mesenchymal Stem Cells Derived from Human Exocrine Pancreas Spontaneously Express Pancreas Progenitor-Cell Markers in a Cell-Passage-Dependent Manner

**DOI:** 10.1155/2016/2142646

**Published:** 2016-08-18

**Authors:** Song Lee, Seonghee Jeong, Chanmi Lee, Jooyun Oh, Song-Cheol Kim

**Affiliations:** ^1^Laboratory of Stem Cell Biology and Cell Therapy, Asan Institute for Life Science, Asan Medical Center, Seoul 05505, Republic of Korea; ^2^Department of Surgery, University of Ulsan College of Medicine and Asan Medical Center, 88 Olympic-ro 43-gil, Songpa-gu, Seoul 05505, Republic of Korea

## Abstract

Mesenchymal stem cells (MSCs) derived from bone marrow, adipose tissue, and most connective tissues have been recognized as promising sources for cell-based therapies. MSCs have also been detected in human pancreatic tissue, including endocrine and exocrine cells. These adult human pancreas-derived MSCs have generated a great deal of interest owing to their potential use in the differentiation of insulin-producing cells for diabetes treatment. In the present study, we isolated MSCs from the adult human exocrine pancreas to determine whether isolated MSCs have the potential to differentiate into pancreatic endocrine cells and, therefore, whether they can be used in stem cell-based therapies. Pancreatic tissue was digested by collagenase and an enriched exocrine-cell fraction was obtained by density-gradient separation. Crude exocrine cells were methodically cultured in suspension and then in adherent culture. We expanded the human pancreatic exocrine-derived MSCs (hpMSCs) by cell passaging in culture and confirmed by flow cytometry that >90% expressed human classic surface markers of MSCs. Interestingly, these cells expressed pancreatic transcription factors, such as Pdx1, Ngn3, and MafA, similar to pancreatic progenitor cells. These results indicated that hpMSCs can be used for the differentiation of pancreatic endocrine cells and may be used in type 1 diabetes treatment.

## 1. Introduction

Currently, there no is cure for diabetes. Although type 2 diabetes, once known as adult-onset or noninsulin-dependent diabetes, can be partially controlled by a healthy diet and regular exercise, type 1 diabetes involves autoimmunity against *β*-cells and can only be treated by transplantation of islet cells or the pancreas [1, 2]. However, after a transplant, recipients must take immunosuppressive medications. Therefore, many researchers are investigating new autologous organ sources of *β*-cells.

Adult stem cells are undifferentiated cells found in very small numbers in many tissues and organs. Adult stem cells can self-renew indefinitely and can differentiate to yield some or all of the major specialized cell types of a tissue or organ [3, 4]. The key functions of adult stem cells in a living organism are to maintain and repair the specific tissues in which they reside [5, 6]. Importantly, adult stem cells are not controversial in research and therapy, because their acquisition does not require destruction of an embryo.

Human mesenchymal stem cells (hMSCs) are a type of adult stem cells that can be easily isolated from several tissues, such as bone marrow [7, 8], adipocytes [8], cord blood [8–10], peripheral blood [10], and the pancreas [11–13], and can be successfully expanded* in vitro*. hMSCs have the ability to self-renew and to differentiate into multiple lineages, including mesodermal lineages, such as adipocytes, chondrocytes, and osteocytes, as well as ectodermal and endodermal cells [14, 15]. hMSCs have several advantages in cell-based clinical therapies, including homing efficiency to damaged tissue sites [16, 17], capability of differentiation into a variety of cell types [18, 19], production of growth factors dependent on environment [20, 21], and effectiveness in treating various immune disorders [22, 23]. Several reports showed that insulin-producing *β*-cells could differentiate from hMSCs [24–27]. Additionally, researchers verified that hMSCs might play a direct role in supporting differentiation into insulin-producing cells or in pancreatic regeneration [28, 29]. These results demonstrated that hMSCs are an important source of cells that can differentiate into pancreatic *β*-cells.

In the present study, we aimed to determine whether MSCs derived from adult human exocrine pancreas (hpMSCs) have the capacity to differentiate into *β*-cells. We hypothesized that isolated tissue-specific stem cells would facilitate differentiation into cells of the identical tissue type. Accordingly, pancreas tissue-derived MSCs may differentiate into pancreatic cells, including endocrine cells, because their gene expression is similar to that of terminally differentiated pancreatic cells. Therefore, we preferentially isolated MSCs from the exocrine pancreas and validated their hpMSC characteristics through analysis of cell-surface phenotype markers by flow cytometry and mRNA expression levels relative to nonpancreatic tissue-derived MSCs by quantitative polymerase chain reaction (qPCR).

## 2. Material and Methods

### 2.1. Isolation of MSCs from Human Exocrine Pancreas

Normal adult pancreatic tissue from five different patients was obtained in compliance with the provisions of the Institutional Review Board of Asan Medical Center, Seoul, Korea, and conducted in accordance with the Declaration of Helsinki and Good Clinical Practice. To separate islet and exocrine cells, a modified Ricordi method of pancreas islet isolation was followed [30]. To obtain pure exocrine cells, islets were separated from digested tissue using continuous-density gradients in OptiPrep solution (Sigma-Aldrich, St. Louis, MO, USA) with a COBE 2991 cell processor (Gambro BCT Inc., Lakewood, CO, USA). The exocrine cells were cultured in suspension in Roswell Park Memorial Institute (RPMI) medium 1640 (Gibco, Carlsbad, CA, USA) supplemented with 10% fetal bovine serum (FBS) and 1% antibiotics in nontissue culture plates at 37°C and 5% CO_2_. Medium was changed after 12 h the first time and then changed every day for 2 days in order to eliminate dying exocrine cells. Suspended exocrine cells were transferred to tissue culture plates for adherent culture in RPMI medium 1640 (Gibco) supplemented with 10% FBS and 1% antibiotics for 4 days. A small number of hpMSCs migrated out from the adherent exocrine cells during early culture. Pancreatic exocrine cells and hpMSCs at a confluency of >80% (4–6 days) were detached with 0.25% Trypsin-EDTA (TE; Invitrogen, Carlsbad, CA, USA) and subcultured in fresh tissue culture plates with Dulbecco's Modified Eagle Medium (Gibco) supplemented with 10% FBS and 1% antibiotics. Adherent hpMSCs were observed using an optical microscope and confirmed morphologies like fibroblast cells.

### 2.2. Flow Cytometry Analysis

hpMSCs were incubated with antibodies against cluster of differentiation (CD) markers and phycoerythrin- (PE-) conjugated monoclonal mouse anti-human CD73, CD90, CD105, CD45, and CD31 antibodies (BD Biosciences, La Jolla, CA, USA). Non-PE-conjugated monoclonal mouse antibodies MCAM, HCAM, CD14, CD19 (Millipore, Billerica, MA, USA), alpha smooth-muscle actin (*α*SMA; Santa Cruz Biotechnology, Dallas, TX, USA), and NCL-L-CA19-9 (Leica Biosystems, Newcastle, UK) were conjugated with anti-mouse IgG-PE (Santa Cruz Biotechnology) or Alexa Fluor 488 anti-mouse IgG (Life Technologies, Carlsbad, CA, USA) after primary incubation. Antibody dilutions and incubation times were those recommended by the manufacturer. Cells were incubated with antibodies at 4°C for 30 min in the dark, and PE-labeled isotype-matched IgG was used as the negative control. Labeled hpMSCs were analyzed on a FACS Caliber flow cytometer (BD Biosciences).

### 2.3. Immunofluorescence Staining

For immunofluorescence staining, slides with cultured hpMSCs were fixed with 4% paraformaldehyde (PFA; Merck, Darmstadt, Germany) for 30 min at 4°C and washed twice with phosphate-buffered saline (PBS). hpMSCs were permeabilized with 0.3% Triton X-100 at 4°C for 5 min and washed three times with PBS. For antibody blocking, hpMSCs were incubated in 5% normal goat serum for 30 min at room temperature (RT). The primary antibody was incubated with anti-rabbit insulin (1 : 200; Santa Cruz Biotechnology), rabbit anti-human glucagon (1 : 100; DaKo, Glostrup, Denmark), anti-rabbit amylase (1 : 200; Sigma-Aldrich), and anti-mouse CA19-9 (1 : 200; Leica Biosystems) in order to confirm endocrine and exocrine cells. Identification of markers associated with hpMSCs was performed using anti-rabbit *α*SMA (1 : 200; Santa Cruz Biotechnology), anti-mouse HCAM, MCAM, CD14, and CD19 (1 : 100; Millipore), and anti-mouse CD90 and CD105 (1 : 100; BD Biosciences). For detection of major pancreatic endocrine transcription factors, we used anti-goat Pdx1 (1 : 10000; Abcam, Cambridge, UK), anti-rabbit Ngn3 (1 : 100; Abcam), and anti-rabbit MafA (1 : 200; Novus Biologicals, LLC, Littleton, CO, USA). All primary antibodies were incubated overnight at 4°C. For secondary fluorescence labeling, hpMSCs were incubated with Alexa Fluor 488 goat anti-mouse or anti-rabbit IgG (1 : 200; Life Technologies) and Alexa Fluor 594 goat anti-rabbit IgG (1 : 200; Life Technologies). Hoechst 33342 (1 : 100; Thermo Scientific, Rockford, IL, USA) was used to stain nuclei for 3 min at RT, and then cells were washed three times with PBS. The slides were visualized under an LSM710 confocal microscope (Carl Zeiss, Oberkochen, Germany).

### 2.4. Reverse-Transcription PCR (RT-PCR)

Total RNA was extracted from hMSCs using Trizol (Invitrogen) according to manufacturer instructions. Total RNA was extracted from exocrine cells at culture days 2 and 4 and from hpMSCs at passage days 2, 4, 6, 8, 10, and 12. cDNA was synthesized from 1 *μ*g RNA template by an oligo-dT primer using a SuperScript III reverse-transcription kit (Invitrogen) at 50°C for 60 min and 70°C for 15 min. PCR reactions were performed using rTaq plus master mix (ELPIS Biotech, Seoul, South Korea) with gene-specific forward and reverse primers (Table 1). PCR amplification conditions were as follows: initial denaturation at 95°C for 5 min, followed by 30 cycles at 95°C for 20 s or 55°C or 57°C for 20 s, and extension at 72°C for 20 s, with a final extension at 72°C for 3 min. RT-PCR results were normalized against the *β*-2 microglobulin housekeeping gene.

### 2.5. qPCR

Real-time PCR products were monitored with a TaqMan probe using AccuPower Plus DualStar qPCR premix (Bioneer, Daejeon, Korea). The TaqMan assays used the 5′- to 3′-exonuclease activity of* Taq* DNA polymerase, and each reaction contained a gene-specific primer and a fluorescence dye-labeled TaqMan probe. The probe contained 5′-reporter dye FAM (6-carboxyfluorescein) and 3′-quencher dye TAMRA (carboxytetramethylrhodamine), and each probe was designed to anneal to the target sequence between the forward and reverse PCR primers. Pancreatic endocrine gene-specific primers were also designed (Table 2). The qPCR program included a two-step reaction, with predenaturation at 95°C for 5 min, denaturation at 95°C for 15 s, and 45 cycles of annealing/extension/detection at 55°C or 60°C for 20 s. After the reaction was completed, gene-expression analyses using the 2^−(ΔΔCt)^ method were performed.

### 2.6. Statistical Analysis

Data were expressed as mean ± standard deviation (SD). Statistical analysis was performed using SigmaPlot 8.0 statistical software (SPSS Inc., Chicago, IL, USA), and a Student *t*-test was set to *p* < 0.05 and *p* < 0.005.

## 3. Results

### 3.1. Distinguishing Features of Adult Human Exocrine Pancreas Cells

**Figure 1:**
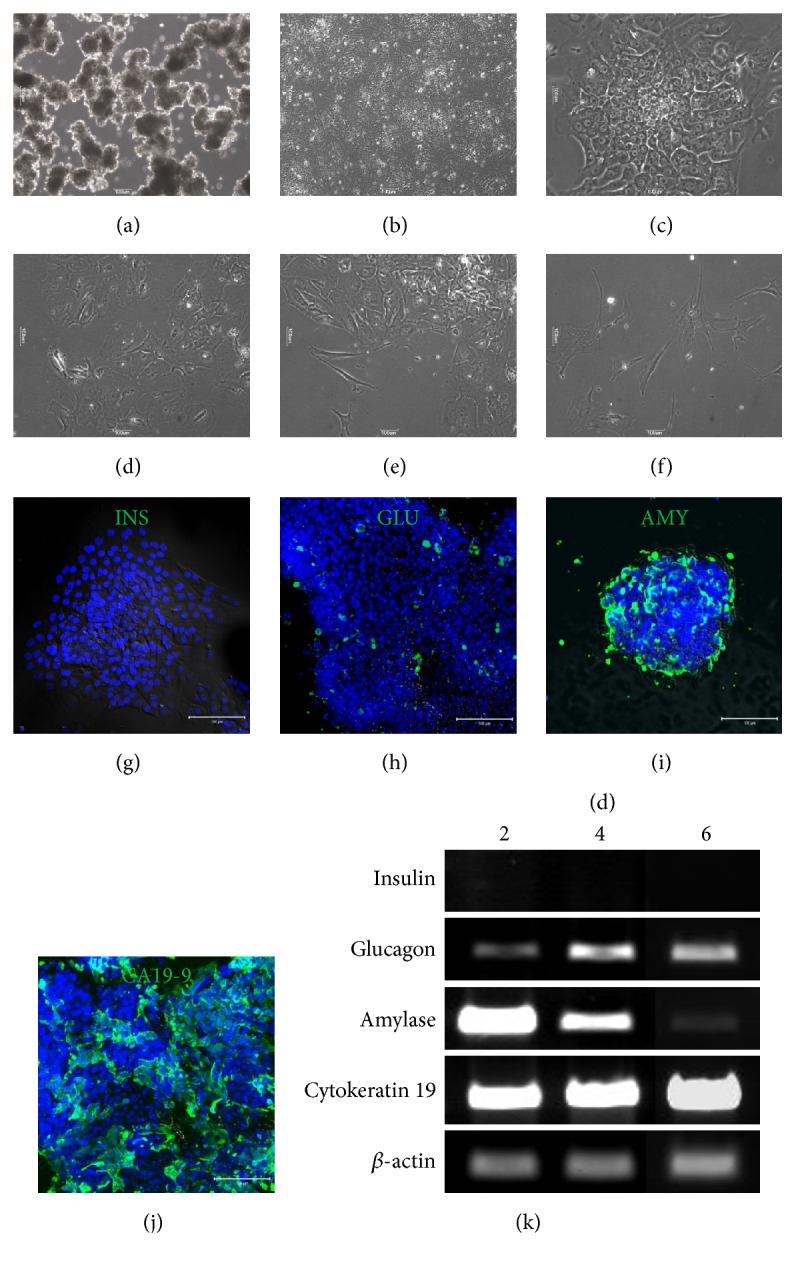
*In vitro* culture of adult human exocrine pancreas cells. (a) Separated exocrine cells from adult pancreas tissue were suspension cultured on nontissue culture plate for 3 days, resulting in aggregation of single exocrine cells into clusters. (b) Exocrine clusters attached to new tissue culture plates rapidly proliferated into a monolayer. (c) After 3 days of monolayer culturing, the majority of exocrine cells displayed epithelial-like morphology and grew in tight clusters. (d) Increasing culture incubation times resulted in morphology changes in some epithelial-like cells; specifically (e) cells located outside of clusters acquired a fibroblast-like morphology and (f) proliferated alongside the epithelial-like cells. (g) Insulin-positive cells were not detected in epithelial-like exocrine cells at culture day 4; however, (h) glucagon-positive cells were detected. (i, j) A majority of exocrine cells exhibited positive-staining results for amylase and CA19-9. (k) Detection of pancreatic cell markers for insulin, glucagon, amylase, and cytokeratin 19 mRNA in exocrine cells on culture days 2, 4, and 6. Amylase mRNA expression decreased over the culture period and was not observed at culture day 6, whereas cytokeratin 19 mRNA expression was continuously detected up to culture day 6.

We contrived a two-step culture method for adult human exocrine pancreas cells in order to collect high-purity exocrine cells. Adult human exocrine pancreas cells were cultured in suspension on nontissue culture plates for 3 days, during which time the cells grew in clusters (Figure 1(a)). After exchanging for a tissue culture plate, exocrine clusters attached to the plate within 2 days, and new exocrine-cell monolayers grew from the exocrine clusters immediately following attachment (Figure 1(b)). These cells showed an epithelial-like cell morphology, with the fastest proliferation based on the monolayer mass occurring during culture day 6 (Figure 1(c)). We also observed that some cells deviated from having epithelial-like morphology in areas of low cell density (Figures 1(d) and 1(e)). These cells proliferated independently and displayed morphology similar to that of fibroblast cells (Figure 1(f)).

For characterization of the attached exocrine clusters, immunofluorescence staining was performed for pancreatic cell markers, such as insulin, glucagon, amylase, and CA19-9. Insulin-positive cells were not detected (Figure 1(g)); however, a few single glucagon-expressing cells were detected on day 4 (Figure 1(h)). Amylase, enzymes secreted from acinar cells, and pancreatic duct-cell marker CA19-9 were mostly detected in exocrine cells on culture day 4 (Figures 1(i) and 1(j)). Gene-expression patterns also showed similar results on culture days 2, 4, and 6 (Figure 1(k)). Insulin mRNA was not expressed; however, glucagon mRNA was expressed weakly at culture days 2, 4, and 6. Additionally, amylase mRNA expression decreased, whereas cytokeratin 19 mRNA was expressed consistently throughout the culture period. These data suggested that our isolated and cultured exocrine cells were generally pure-grade cells without endocrine cells.

### 3.2. Expansion of MSCs from Exocrine Cells and Phenotype Validation

**Figure 2:**
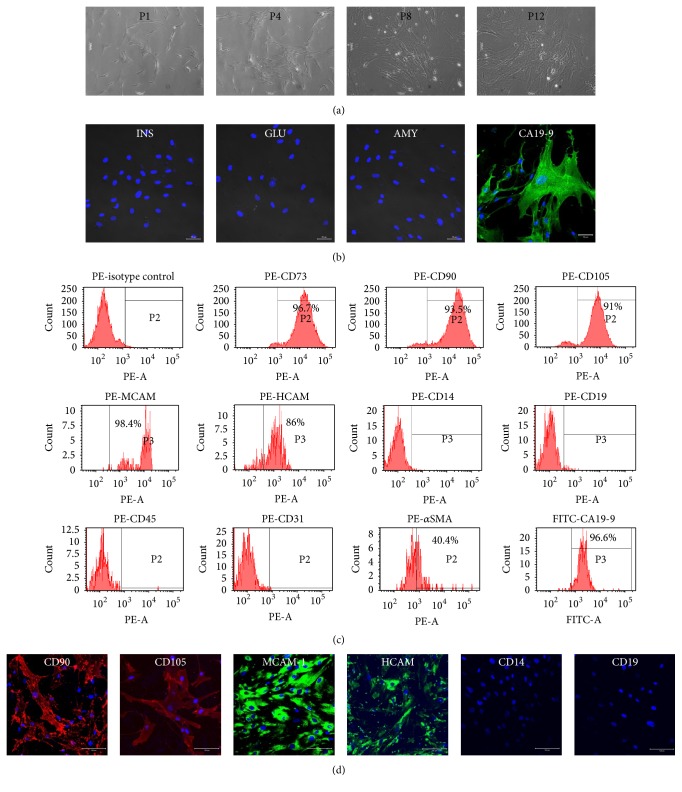
Phenotypic characteristics of expanded MSCs. (a) Cells with fibroblast-like morphology attached to the culture plate after the first passage of exocrine cells and maintained a similar morphology throughout continuous passages 1 through 12. (b) Passage six hpMSCs were fixed with 4% PFA and underwent immunofluorescence staining with anti-insulin, glucagon, amylase, and CA19-9. Pancreatic cell hormone insulin, glucagon, amylase, and enzymes from exocrine cells were not detected, whereas CA19-9 was detected in most cells. (c) Flow cytometry analysis of fibroblast-like cells harvested at passage day 6. hMSC markers were labeled using PE-conjugated antibodies against isotypes CD73, CD90, CD105, CD14, CD19, CD45, CD31, MCAM, HCAM, *α*SMA, or FITC-conjugated CA19-9. hMSC-positive markers CD73 (96.7%), CD90 (93.5%), CD105 (91%), *α*SMA (40.4%), MCAM (98.4%), and HCAM (86%) were detected, and hMSC-negative markers CD14, CD19, CD45, and CD31 were not. Pancreatic duct cells positive for CA19-9 (96.9%) were also detected. (d) hMSC-phenotype markers confirmed by immunofluorescence staining. CD90, CD105, MCAM, and HCAM exhibited the strongest signals, whereas CD14 and CD19 were not detected during passage six.

**Figure 3:**
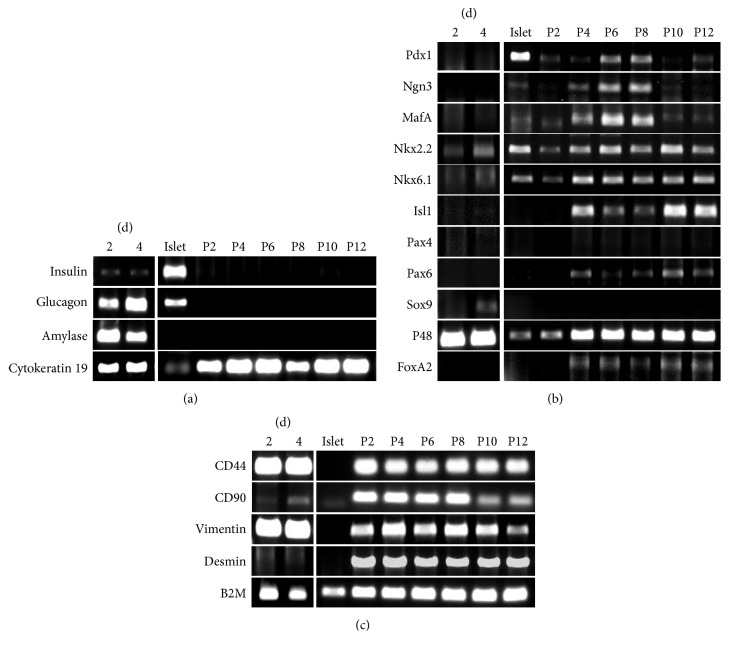
Pancreatic progenitor markers expressed in hpMSCs. RT-PCR analysis of mRNA expression in exocrine cells, hpMSCs, and control human islets. Exocrine mRNA was extracted at culture days 2 and 4, and hpMSC mRNA was isolated from cells on passage days 2, 4, 6, 8, 10, and 12. (a) Cytokeratin 19 was expressed in all passages of hpMSCs, whereas insulin, glucagon, and amylase were not. (b) Pancreatic cell transcription factors, including those required for development and differentiation, exhibited different expression levels depending on passage. Key transcription factors Pdx1, Ngn3, and MafA were detected from passages four through eight in culture cells; Nkx2.2, Nkx6.1, and P48 were expressed in all hpMSCs without being affected by cell passage, and Isl1 and Pax6 expression was initiated during passage four. Weak expression of Nkx2.2 and Nkx6.1 and strong expression of the P48 exocrine-cell marker were detected in exocrine cells. (c) hpMSCs continue to display markers associated with hpMSCs, including CD44, CD90, vimentin, and desmin.

To expand hpMSCs, primary exocrine clusters were cultured until cells covered the entire plate. However, only a small number of hpMSCs grew as compared with the growth of exocrine cells during the primary culture. hpMSCs were starting to emerge after the first passage of exocrine cells, during which time almost all epithelial-like exocrine cells had not attached to the new tissue culture plate; however, fibroblast-like cells (hpMSCs) attached and grew easily. hpMSC expansion gradually increased with continuous passages (Figure 2(a)), and while adult pancreatic cell markers, such as insulin, glucagon, and amylase, were not detected, CA19-9 was expressed during passage four (Figure 2(b)). To confirm the presence of specific hMSC markers, cell-surface antigen expression on hpMSCs after four passages was analyzed by immunofluorescence staining and flow cytometry (Figures 2(b) and 3). Antigens for CD73, CD90, CD105, MCAM, and HCAM cell-surface markers associated with hMSCs were expressed in ≥85% of hpMSCs (Figures 2(c) and 2(d)). These cells did not, however, express the hMSC-negative markers CD14, CD19, CD45, or CD31, indicating that these cells exhibited an hpMSC phenotype (Figures 2(c) and 2(d)). *α*SMA, a minor marker of hMSCs, was also expressed in hpMSCs, and, interestingly, the pancreatic duct-cell marker CA19-9 was expressed in most cells (Figure 2(c)). This phenotype indicated that hpMSC isolation and expansion from pure pancreatic exocrine cells were possible without special separation methods. However, hpMSCs simultaneously expressed both the pancreatic duct-cell marker CA19-9 and hMSC markers, suggesting that hpMSCs may retain a pancreatic duct-cell phenotype after hpMSC transformation.

### 3.3. hpMSC Expression of Pancreas Progenitor Genes

**Figure 4:**
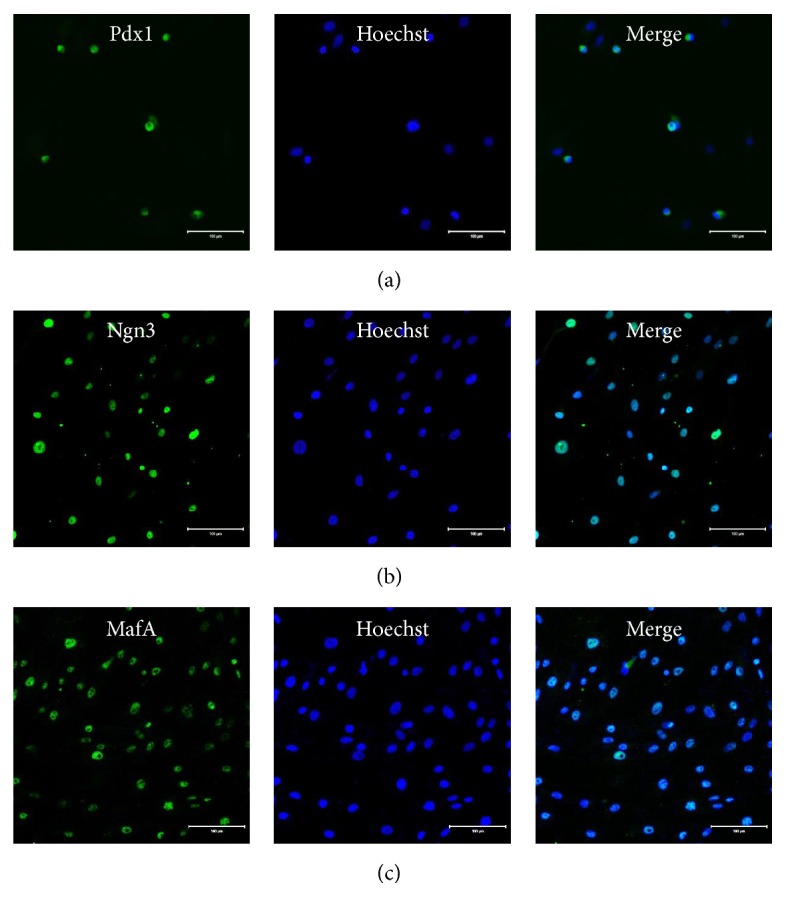
Key pancreatic transcription factors expressed in hpMSCs. Pdx1, Ngn3, and MafA are key transcription factors involved in pancreas development and pancreas progenitor-cell differentiation into *β*-cells. (a) Pdx1-, (b) Ngn3-, and (c) MafA-positive immunofluorescence staining results in the nucleus of hpMSCs during passage 6. Scale bar = 100 *μ*m.

To evaluate mRNA expression in exocrine cells and hpMSCs, cells were analyzed by RT-PCR for expression of genes known to be expressed differentially by MSCs and pancreatic cells, including developed and progenitor cells. Insulin and glucagon mRNA, mature pancreatic endocrine-cell hormones, amylase mRNA, and mature exocrine-cell digestive enzymes were not expressed in hpMSCs at passages 2, 4, 6, 8, 10, and 12 but were expressed in crude exocrine and control human islet (hIslet) cells. Pancreatic duct-cell marker cytokeratin 19 mRNA was detected in exocrine cells, islet cells, and all passages of hpMSCs (Figure 3(a)). We also analyzed pancreatic progenitor cells and differentiation markers, such as Pdx1, Ngn3, MafA, Nkx2.2, Nkx6.1, Isl1, Pax4, Pax6, Sox9, P48, and FoxA2 (Figure 3(b)). Interestingly, Pdx1, Ngn3, MafA, Nkx2.2, Nkx6.1, Isl1, Pax6, and P48 mRNA expression was altered in passaged hpMSCs, whereas crude exocrine cells exhibited decreased expression of Nkx2.2 and Nkx6.1 and increased expression of P48. Expression of the major transcription factors in the pancreas, represented by Pdx1, Ngn3, and MafA mRNA, was detected from hpMSC passage four but gradually disappeared from passage 10. However, Nkx2.2 and Nkx6.1 mRNA expression was detected during the entire hpMSC subculture period. The mRNA expression levels of early endocrine-cell markers Islet-1 (Isl1), Pax6, and FoxA2 were initially detected at hpMSC passage four and were consistently detected at passage 12. Expression of other endocrine-cell differentiation markers, such as Pax4 and Sox9, was not detected during the continuous passage of hpMSCs. The hMSC markers CD44, CD90, vimentin, and desmin were expressed in all passaged hpMSCs; however, CD90 and desmin were not expressed or expressed at low levels in crude exocrine cells (Figure 4(c)). These results indicated successful isolation of hpMSCs having similar characteristics as hMSCs, while simultaneously expressing pancreatic progenitor markers.

### 3.4. Spontaneous Expression of Pdx1, Ngn3, and MafA in hpMSCs

**Figure 5:**
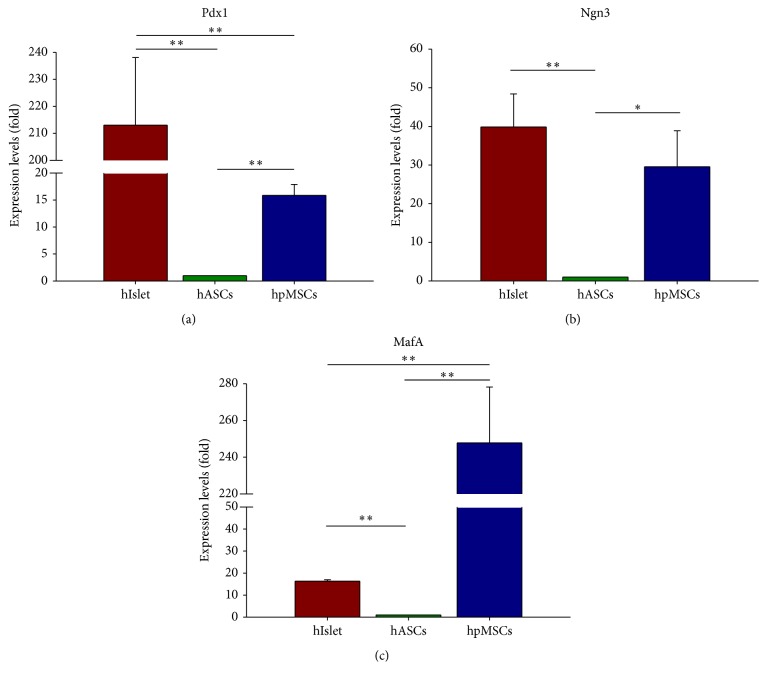
Quantitative analysis of mRNA expression of key pancreatic transcription factors in hpMSCs. Analysis of key pancreatic transcription factors Pdx1, Ngn3, and MafA expression in human islet cells, human ASCs, and hpMSCs by qPCR. Reference expression levels (1-fold) were calculated relative to baseline expression levels measured in hASCs. (a) Pdx1 was highly expressed in islet cells (213.040 ± 25.002) and hpMSCs (15.867 ± 2.019), whereas (b) Ngn3 was expressed in both islet cells (39.855 ± 8.529) and hpMSCs (29.535 ± 9.319) at comparable levels. (c) MafA was expressed at higher levels in hpMSCs (247.826 ± 30.376) than in islet cells (16.342 ± 0.640). Error bars indicate SD (*n* = 3); ^*∗*^ *p* < 0.05; ^*∗∗*^ *p* < 0.005.

We confirmed expression of major pancreatic progenitor-cell transcription factors Pdx1, Ngn3, and MafA during passage six of hpMSCs by immunofluorescence staining. Pdx1 was comparatively detected with overlapping nuclei in hpMSCs (Figure 4(a)), whereas both Ngn3 and MafA were generally detected in hpMSCs (Figures 4(b) and 4(c)). We also compared the expression of these key transcription factors between pancreatic islet cells, human adult adipose tissue-derived mesenchymal stem cells (hASCs), and hpMSCs in order to define hpMSC characteristics. The mRNA expression levels of Pdx1, Ngn3, and MafA were analyzed by qPCR, and the relative expression levels calculated based on their expression in hASCs. hIslet cells exhibited higher levels of Pdx1 expression than those in hASCs; however, Pdx1 expression in hpMSCs was 15-fold higher than that observed in hASCs (Figure 5(a)). Additionally, both hIslet cells and hpMSCs displayed higher levels of Ngn3 expression than those observed in hASCs (Figure 5(b)), whereas MafA mRNA expression levels were higher in hpMSCs than in hIslet cells (Figure 5(c)). These results indicated that hpMSCs spontaneously expressed key pancreatic transcription factors similar to pancreatic progenitor cells.

## 4. Discussion

In this study, we isolated hpMSCs from human adult pancreatic exocrine tissue and expanded the cell population* in vitro* using a new method. Because the isolation of MSCs is simple and does not require any special technique and because MSCs easily attach to culture plates and show robust proliferation, they have been widely isolated from different tissues. However, we consider that the source of the MSCs is important because the isolated MSCs share some degree of their gene-expression characteristics with the origin tissue and also have lineage-specific differentiation ability.

Human pancreatic cells, such as ductal epithelial-, islet-, or exocrine-cell-derived MSCs, were reported to exhibit characteristics of differentiated insulin-producing *β*-cells [12, 13, 31, 32]. However, insulin-producing *β*-cells derived from MSCs isolated from other tissue, such as bone marrow, adipose, and Wharton's jelly, were induced by reprogramming gene induction/transfection [33–35]. These reprogramming methods may lead to immediate and effective differentiation into *β*-cells but may not constitute a suitable method of cell transplantation during clinical trials of therapies for the treatment of diabetes, because exogenous genes may prove unsafe due to ectopic expression and instability. We confirmed that hpMSCs expressed key transcription factors involved in the differentiation to insulin-producing cells, depending on the cell passage. This endogenous expression is preferable to exogenous expression mediated by viral gene delivery [33–35]. However, the endogenous expression also has some potential disadvantages in that the expression of the transcription factors is not controllable; thus, it may not be sufficiently constitutive or transient for the desired use of the cells.

Therefore, we developed a new method to isolate and expand hpMSCs expressing the key transcription factors required of pancreatic progenitor cells to form adult human pancreatic exocrine cells. Our method has the advantage of using adult pancreatic tissue rather than fetal pancreas [11]. Although fetal pancreatic cells have higher cell proliferation activity and differentiation capacity than adult pancreatic cells, they are difficult to obtain and not able to be clinically applied because they are not autologous. In this study, we did not try to separately culture pancreatic ductal epithelium cells [12]. These cells were present in the primary exocrine-cell culture, but they never attached to the tissue culture plate and were thus removed during the first passage. Therefore, we were able to increase the purity of the hpMSCs without performing the complicated step of separately removing the duct cells.

For the isolation of hpMSCs from exocrine cells, we used a simple two-step culture method. Since there are a small number of hpMSCs in primary crude exocrine-cell extracts, we preferentially increased the number of primary exocrine cells and simultaneously removed dead cells using suspension culture. During suspension culture, exocrine cells formed spherical clusters, with most clustered cells being positive for the expression of pancreatic amylase and CA19-9 (Figures 1(i) and 1(j)). After transferring to cell culture plates, exocrine clusters attached to the bottom of the plates and epithelial-like cells grew out from the adhesion cluster. After cell passage following TE treatment, most epithelial-like cells were unable to attach to the new tissue culture plates; however, those that did attach exhibited morphology similar to fibroblast-like cells (Figure 2(a)). Additionally, these fibroblast-like cells were negative for pancreatic cell markers, such as insulin, glucagon, and amylase (Figures 2(b) and 3(a)).

We characterized the phenotype of these fibroblast-like cells by flow cytometry (Figure 2(c)), immunocytochemistry (Figure 2(d)), and mRNA amplification using PCR (Figure 3(c)). Our results indicated that these cells were a type of hMSC that continually expressed the pancreatic ductal-cell markers CA19-9 and cytokeratin 19 (Figures 2(b) and 3(a)). We also attempted to differentiate these cells into adipogenic and osteogenic cells from hpMSCs but observed no definitive differentiation into mesoderm lineages (data not shown). One explanation may be the weak mesoderm-differentiation ability of hpMSCs due to their continuous expression of epithelial-cell markers, such as cytokeratin 19 and CA19-9. However, these features may confer an advantage toward differentiating into pancreatic cells, including endocrine cells. Other groups also reported that cytokeratin 19-expressing MSCs could be isolated from human exocrine cells and differentiated into insulin-expressing cells [12, 13] or CA19-9-positive duct cells in human exocrine cells induced with insulin-containing aggregates [36]. This suggested that human pancreas exocrine-derived MSCs expressing epithelial-cell markers could effectively differentiate into endocrine cells, including insulin-expressing cells.

We confirmed endocrine gene expression continuously during the culture of exocrine cells and of hpMSCs (Figure 3(a)). Insulin was weakly expressed and glucagon was strongly expressed by crude exocrine cells, but endocrine gene expression was not detected in hpMSCs. We believe that the expression of these genes in the crude exocrine cells can be attributed to contamination by a small number of endocrine cells because the crude exocrine cells comprised the remaining fraction after islet isolation from the human pancreas. Ultimately, as described above, these endocrine cells died or were removed during passaging and culture.

During characterization of mRNA expression in hpMSCs, we observed expression of several transcription factors related to endocrine-cell development. We observed mRNA expression of Pdx1, Ngn3, and MafA, which are major transcription factors required for the development of all pancreatic endocrine-cell types in hpMSCs (Figures 3(b), 4(a)–4(c), and 5(a)–5(c)). In pancreatic progenitor cells, many transcription factors form mutual networks and differentiate into both exocrine and endocrine cells. In particular, the combination of Pdx1, Ngn3, and MafA is acknowledged as being a logical gene set capable of stimulating pancreatic endocrine development [37, 38]. During development or differentiation of pancreatic cells, Pdx1 is required for definitive endoderm [39] and pancreatic bud development [40] and *β*-cell maturation [41], whereas Ngn3 is required for endocrine precursor cell formation [42] and MafA is required for *β*-cell maturation [43]. Recent studies showed that adenovirus vector-mediated overexpression of Pdx1, Ngn3, and MafA in acinar lineage-converted cells induced islet-like structures [38, 44]. We also compared the relative expression levels of key transcription factors between hIslet cells, hASCs, and hpMSCs by qPCR, with mRNA expression levels in hASCs used as the baseline reference. These results revealed that hpMSCs express the three key transcription factors at higher levels than those observed in hASCs; in particular, MafA expression was higher than that observed in hIslet cells (Figures 5(a)–5(c)). These data indicated that hpMSCs endogenously express Pdx1, Ngn3, and MafA in a cell-passage-dependent manner. hpMSCs from exocrine cells have largely not been studied thus far. The hpMSCs generated by our method expressed various transcription factors related to the differentiation of insulin-producing cells such as Pdx1, Ngn3, MafA, NKx2.2, Nkx6.1, and Isl1. Other hpMSCs isolated from the pancreatic ductal epithelium did not show expression of differentiation-related transcription factors prior to exposure to differentiation conditions, but expression of Pdx1, NeuroD, Ngn3, and Pax4 was detected after differentiation [12].

**Figure 6:**
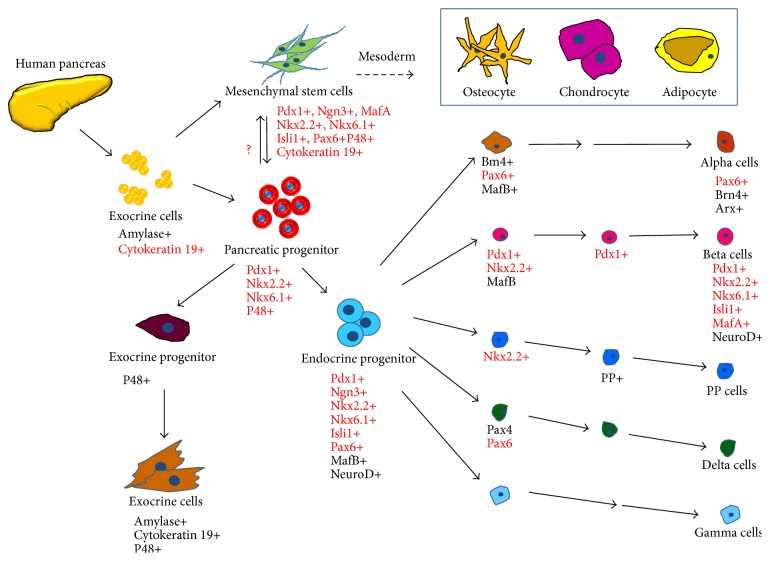
Correlations between transcription factor expressions in hpMSCs and in pancreas progenitor cells. Human exocrine pancreas tissue-derived hpMSCs express Pdx1, Nkx2.2, Nkx6.1, and P48 at levels comparable to those in pancreatic progenitor cells. hpMSCs also specifically express other factors, including Ngn3, Isl1, Pax6, and MafA, associated with pancreas-endocrine developmental lineage.

Here, we confirmed that hpMSCs expressed transcription factors associated with lineage development of pancreatic progenitor cells [45–47] (Figure 6) and specifically described the transcription factors observed in this study that are involved in the pancreas-developmental process. Furthermore, we isolated hpMSCs exhibiting characteristics associated with pancreas progenitor cells, as well as those indicating an ability to differentiate into endocrine cells. Our results suggest that hpMSCs are capable of differentiating into insulin-producing *β*- and endocrine cells under specific culture conditions without the need to externally induce the transduction/transfection of key developmental transcription factors. Although further study is necessary, our findings indicated that hpMSCs can be used as sources of autologous transplantation for the treatment of diabetes.

## Figures and Tables

**Table 1:** Primers used for PCR amplification.

Gene		Sequence (5′ → 3′)	*T*_*m*_ (°C)	Product size (bp)
Insulin	Forward	CATGGCCCTGTGGATGCGC	64	331
	Reverse	AGTTGCAGTAGTTCTCCAG	52.5	

Glucagon	Forward	CCCAAGATTTTGTGCAGTGGTT	57.5	221
	Reverse	GCGGCCAAGTTCTTCAACAAT	57.7	

Amylase	Forward	GGTTCAGGTCTCTCCACCAA	53.5	214
	Reverse	TCCTGCACTCACAGCGTTAC	53.4	

Cytokeratin 19	Forward	AGGTGGATTCCGCTCCGGGCA	68.2	460
	Reverse	ATCTTCCTGTCCCTCGAGCA	55.8	

Pdx1	Forward	GCCCCCGCCGCCGCCGCCGCA	75.1	231
	Reverse	GGCAGCTGGACGCGGTTGGGC	71.2	

Ngn3	Forward	GTAGAAAGGATGACGCCTCAACC	57.5	241
	Reverse	TCAGTGCCAACTCGCTCTTAGG	58.1	

MafA	Forward	TTCAGCAAGGAGGAGGTCAT	53.3	216
	Reverse	CGCCAGCTTCTCGTATTTCT	53.6	

Nkx2.2	Forward	TTCTACGACAGCAGCGACAACC	58.9	393
	Reverse	CGTCACCTCCATACCTTTCTCG	57.2	

Nkx6.1	Forward	ACACGAGACCCACTTTTTCCG	57.6	336
	Reverse	TGCTGGACTTGTGCTTCTTCAAC	58	

Isl1	Forward	ATTTCCCTATGTGTTGGTTGCG	57.4	229
	Reverse	CGTTCTTGCTGAAGCCGATG	58	

Pax4	Forward	TTTGTGCTGAAGGGCTTTGC	57.5	216
	Reverse	GGGAGAAGATAGTCCGATTCCG	57.6	

Pax6	Forward	CGGCAGAAGATTGTAGAG	44.8	290
	Reverse	GATGACACGCTTGGTATG	45.8	

Sox9	Forward	TACGACTACACCGACCACCA	53.4	213
	Reverse	TCAAGGTCGAGTGAGCTGTG	53.3	

P48	Forward	CTGGAATGCCTGGACTCAAT	53.6	232
	Reverse	AGGTTGCTTTTCCTTCAGCA	53.8	

CD44	Forward	AAGGTGGAGCAAACACAACC	53.6	151
	Reverse	AGCTTTTTCTTCTGCCCACA	53.8	

CD90	Forward	CTAGTGGACCAGAGCCTTCG	53.7	236
	Reverse	TGGAGTGCACACGTGTAGGT	53.4	

Vimentin	Forward	GAGAACTTTGCCGTTGAAGC	53.8	170
	Reverse	TCCAGCAGCTTCCTGTAGGT	53.6	

Desmin	Forward	AAGCTGCAGGAGGAGATTCA	53.7	240
	Reverse	GGCAGTGAGGTCTGGCTTAG	53.6	

Beta actin	Forward	AGAGCTACGAGCTGCCTGAC	53.5	184
	Reverse	AGCACTGTGTTGGCGTACAG	53.4	

**Table 2:** Primers and probes used for qPCR amplification.

Gene		Sequence (5′ → 3′)	*T*_*m*_ (°C)	Product size (bp)
Pdx1	Forward	GCATCCCAGGTCTGTCTTCT	60.7	140
	Reverse	CACTGCCAGAAAGGTTTGAA	58.5	
	Probe	AACCCGAGCACAAGGCCCAG	62.9	3′TAMRA, 5′FAM

Ngn3	Forward	GAAAGGACCTGTCTGTCGCT	59	124
	Reverse	AGGGAGAAGCAGAAGGAACA	57.7	
	Probe	CTCCCGGCTCCCTCCCTCTC	62.9	3′TAMRA, 5′FAM

MafA	Forward	TTCAGCAAGGAGGAGGTCAT	60.3	119
	Reverse	TTCTCGCTCTCCAGAATGTG	61.5	
	Probe	CTCAAGCAGAAGCGGCGCAC	62.7	3′TAMRA, 5′FAM

B2M	Forward	GACTTTGTCACAGCCCAAGA	59.2	111
	Reverse	CAAGCAAGCAGAATTTGGAA	61.8	
	Probe	TCATCCAATCCAAATGCGGCA	62.7	3′TAMRA, 5′FAM
